# Assessment of Stress Tolerance, Productivity, and Forage Quality in T_1_ Transgenic Alfalfa Co-overexpressing *ZxNHX* and *ZxVP1-1* from *Zygophyllum xanthoxylum*

**DOI:** 10.3389/fpls.2016.01598

**Published:** 2016-10-27

**Authors:** Peng Kang, Ai-Ke Bao, Tanweer Kumar, Ya-Qing Pan, Zhulatai Bao, Fei Wang, Suo-Min Wang

**Affiliations:** State Key Laboratory of Grassland Agro-ecosystems, College of Pastoral Agriculture Science and Technology, Lanzhou UniversityLanzhou, China

**Keywords:** transgenic alfalfa, stress resistance, nutritive value, phosphorus deficiency, field trial

## Abstract

Salinization, desertification, and soil nutrient deprivation are threatening the production of alfalfa (*Medicago sativa* L.) in northern China. We have previously generated T_0_ transgenic alfalfa co-overexpressing *Zygophyllum xanthoxylum ZxNHX* and *ZxVP1-1* genes with enhanced salt and drought tolerance. To further develop this excellent breeding material into the new forage cultivar, stress tolerance, productivity, and forage quality of T_1_ transgenic alfalfa (GM) were assessed in this study. The GM inherited the traits of salt and drought tolerance from T_0_ generation. Most importantly, co-overexpression of *ZxNHX* and *ZxVP1-1* enhanced the tolerance to Pi deficiency in GM, which was associated with more Pi accumulation in plants. Meanwhile, T_1_ transgenic alfalfa developed a larger root system with increased root size, root dry weight and root/shoot ratio, which may be one important reason for the improvement of phosphorus nutrition and high biomass accumulation in GM under various conditions. GM also accumulated more crude protein, crude fiber, crude fat, and crude ash than wild-type (WT) plants, especially under stress conditions and in the field. More interestingly, the crude fat contents sharply dropped in WT (by 66-74%), whereas showed no change or decreased less in GM, when subjected to salinity, drought or low-Pi. Our results indicate that T_1_ transgenic alfalfa co-overexpressing *ZxNHX* and *ZxVP1-1* shows stronger stress tolerance, higher productivity and better forage quality. This study provides a solid foundation for creating the alfalfa cultivars with high yield, good quality and wide adaptability on saline, dry, and nutrient-deprived marginal lands of northern China.

## Introduction

Salinity, drought, and soil nutrient deprivation, which cause land degradation (Mbarki et al., 2016), are primary limiting factors in plant growth and agricultural productivities. In recent years, due to global climate change and excessive human activities, the soil salinization, desertification, and soil erosion have showed an intensifying trend in northern China. This situation is resulting in a dramatic increase of saline, arid and nutrient-deprived marginal lands in this region. For maintaining the security of ecological system and agriculture in northern China, reclamation and restoration of marginal lands have been one of most urgent issues at present.

Alfalfa (*Medicago sativa* L.) is a perennial legume forage throughout the world. High biomass production, high nutritional quality and widespread adaptability have made alfalfa as a leading forage crop with a good fame of ‘Queen of the Forages’ (Bao et al., 2009; Kumar, 2011). As an economically important legume forage crop, alfalfa has brought considerable profits for local people and companies in northern China. However, the limited arable land resources are the major barrier for further development of alfalfa production in these areas (Bao et al., 2016). This challenge would be readily overcome if a large number of marginal lands were used for alfalfa planting. Paradoxically, most of the existing alfalfa cultivars are difficult to be planted and grow on saline and arid marginal lands, because of weak tolerance to salinity and drought (Maas and Hoffman, 1977; Peel et al., 2004; Kumar, 2011; Bao et al., 2016). In addition to salt and drought stress, inorganic phosphate (Pi) deficiency in soil is another limiting factor that affects the yield and persistence of alfalfa (Berg et al., 2009). Previous studies showed that soil Pi deficiency are common in areas (including northern China) growing alfalfa and other legume crops (Wang et al., 1998; MacDonald et al., 2011; Ma et al., 2012; Bargaz et al., 2016). In the past decades, farmers have to counter this problem through the application of phosphate fertilizer (Kochian et al., 2004; Gaxiola et al., 2011, 2012); however, this process resulted in the increase of cost and damage of environment (Giaveno et al., 2010; Gaxiola et al., 2011, 2012; Pei et al., 2012; Yang et al., 2014; Lv et al., 2015). Therefore, developing alfalfa cultivars suitable for growing on saline, arid and nutrient-deprived marginal lands is necessary to promote alfalfa production and provide substantial environmental benefits in northern China.

Previous studies have demonstrated that tonoplast Cation/H^+^ antiporters (NHXs) and H^+^-pyrophosphatase (H^+^-PPase) play important roles in a series of physiological and biochemical processes including vacuolar compartmentation of Na^+^, intracellular ions and pH homeostasis, stomatal movements, water uptake, plant development, nutrient use efficiency, and transport of photosynthates (e.g., Bassil et al., 2011; Ferjani et al., 2011, 2012; Gaxiola et al., 2012; Andrés et al., 2014; Bassil and Blumwald, 2014; Reguera et al., 2014; Pizzio et al., 2015; Khadilkar et al., 2016). Overexpression of NHXs or H^+^-PPase genes significantly improved growth performance and the tolerance to multiple abiotic stresses in various transgenic plants (e.g., Apse et al., 1999; Gaxiola et al., 2001; Zhang and Blumwald, 2001; Park et al., 2005; Schilling et al., 2014; Yang et al., 2014). Of significance, co-overexpression of both NHXs and H^+^-PPase conferred transgenic plants greater tolerance and higher biomass accumulation than expression of the single gene (Zhao et al., 2006; Liu et al., 2010; Bhaskaran and Savithramma, 2011; Gouiaa et al., 2012; Bao et al., 2014). These findings indicated that tonoplast NHXs and H^+^-PPase genes have the potential in the development of crop cultivars with stronger stress tolerance, higher yield and better quality.

In previous study, we co-overexpressed two xerophyte genes, *ZxNHX* and *ZxVP1-1* from *Zygophyllum xanthoxylum*, encoding vacuolar membrane NHX and H^+^-PPase, respectively, in alfalfa. The T_0_ transgenic plants co-overexpressing *ZxNHX* and *ZxVP1-1* show improved growth performance and enhanced tolerance to salinity and drought (Bao et al., 2016). However, previous work only tested the salt and drought tolerance in T_0_ generation. To develop this excellent breeding material into a new forage cultivar, it is necessary to investigate if transgenic progeny could stably inherit excellent traits from T_0_ generation. Therefore, in the present study, we performed a comprehensive evaluation on T_1_ generation transgenic alfalfa through investigating its stress tolerance, productivity and forage quality in the greenhouse and under field conditions. The T_1_ transgenic alfalfa inherited the excellent traits from T_0_ generation and exhibited stronger stress tolerance (to salinity, drought and phosphate deficiency), higher biomass accumulation and forage quality, compared to wild-type (WT) plants.

## Materials and Methods

### Characterization of T_1_ Transgenic Alfalfa Plants

The seeds harvested from T_0_ transgenic alfalfa L9 line (Bao et al., 2016) were germinated in 1/2 MS medium containing 50 mg/l hygromycin for 2 weeks, then six of surviving plants were randomly chosen for molecular characterization. The PCR analysis was conducted using genomic DNA isolated from leaf of putative T_1_ transgenic plants and WT plants according to the method as described by Bao et al. (2014).

To perform further molecular and physiological assays, the first PCR positive plant and WT were propagated from stem cuttings as described by Bao et al. (2014, 2016). Then total RNA was extracted from root, stem and leaf of propagated transgenic plants (GM) and WT with a Trizol Kit (Sangon Biotech, Shanghai, China) following manufacturer’s instructions. The primers, procedures, and conditions of PCR and RT-PCR analyses for *ZxVP1-1* and *ZxNHX* genes were the same as previous report (Bao et al., 2016).

### Salt, Drought, and Phosphate (Pi) Deficiency Experiments in the Greenhouse

For salt treatment experiment, the uniform plants of GM and WT were transplanted into separate plastic cylindrical pots (8 cm diameter × 10 cm high with a five-mm-diameter small hole at the bottom, one plant per pot) containing vermiculite and perlite (1:1) under a photoperiod of 16/8 h (light/dark, the light density during the light period was 800 mmol/m^2^/s) at 26 ± 2°C and 60 ± 5% of relative humidity (RH). The pots were placed in the plastic rectangular trays (40 cm × 50 cm, 20 pots per tray). Two liters of 1/2 strength Hoagland nutrient solution was poured into each tray and changed (with fresh nutrient solution) every 2 days to culture the plants for 4 weeks, then NaCl was added into the nutrient solution and increased with 50 mM/day to 200 mM. After salt treatment for 20 days, the plants were harvested for further assessment. The plants from same scheme but without irrigation of NaCl solution were used as control.

For drought treatment experiment, T_1_ transgenic plants and WT were transplanted into plastic cylindrical pots (the same as which in salt treatment, one plant per pot) filled with 300 g oven-dried artificial soil with a mixture of vermiculite, perlite, and peat moss (v/v, 1:1:1), and watered with 1/8 strength Hoagland nutrient solution. The soil water content was controlled at 70% of field water capacity (FWC, the absolute soil water content at FWC is 2.5 g/g) by weighing every day for 4 weeks. After that, the plants from GM and WT were divided into two groups, respectively: control and drought treatment. The soil water content was maintained at 70% of FWC in the control group, while it was reduced to 30% of FWC in the drought group. After 20 days of drought treatment, plants were harvested for further analysis. The growth conditions during the experimental period were the same as that in salt treatment experiment.

For Pi deficiency treatment (low-Pi) experiment, the GM and WT plants were transplanted into separate plastic cylindrical pots (the same as which in salt treatment, one plant per pot) containing perlite (the main constituent is the quartz and doesn’t contain Pi). The pots were placed in plastic rectangular trays (40 cm × 50 cm, 20 pots per tray). Two liters of 1/2 strength Hoagland nutrient solution containing 0.5 mM NH_4_H_2_PO_4_ (control) or reduced NH_4_H_2_PO_4_ concentration to 5 μM (low-Pi, the reduction of N was supplied with NH_4_NO_3_) was poured into each tray and changed (with corresponding fresh nutrient solution) every 2 days to culture the plants for 20 days. The growth conditions during the experimental period were the same as that in salt treatment experiment.

### Determination of Plant Growth

At the end of treatments, the shoot height and root length were measured by a flexible rule. The root volume was determined according to the method described by Musick et al. (1965). After that, the plants were dried in an oven at 80°C for 72 h and the total dry weight (DW) were determined.

### Measurement of Total P Concentration

Total P concentrations were determined according to the method described by Pei et al. (2012) with minor modification. Briefly, plants were divided into leaves and roots and then were dried in an oven at 80°C for 72 h. After measuring the DWs, the samples were ashed in a Muffle furnace (TNX1700-30; Shinbae Industrial Co. Ltd, Shanghai, China) at 600°C for 6 h. The ash samples were dissolved in 10 ml 1 M HCl and a few drops of HNO_3_. The Pi concentration was determined using a spectrophotometer (UV-6100PCS; Mapada Instruments Co. Ltd, Shanghai, China).

### Measurement of Crude Protein (CP), Crude fiber(CFI), Crude Fat(CF), and Crude Ash (CA)in Shoot

The shoot samples from T_1_ transgenic alfalfa and WT plants were oven-dried for 72 h at 80°C and ground to pass a 1.0 mm screen. The contents of crude protein (CP), crude fibre (CFI), crude fat (CF), and crude ash (CA) were analyzed according to official methods from the National Standards of P.R. China (GB/T 6432-94 for CP, SN/T 0800.8-1999 for CFI, GB/T 6433-2006 for CF, and GBT 6438-2007 for CA, respectively).

### Assessment of Productivity and Forage Quality of T_1_ Transgenic Alfalfa in the Field Conditions

The location of the field trial is Yuzhong Experimental Station of Lanzhou University, of which detailed information were reported previously (Bao et al., 2016). The soil available nitrogen, phosphate and potassium in our experiment field were 4.1, 0.8, and 4.9 μmol/g dry soil, respectively, where the relative low available P content is actually the main limiting factor to growth of alfalfa. T_1_ transgenic alfalfa and WT were firstly cultured for 60 days in greenhouse, then the shoots were trimmed away and the remaining plants with 5 cm stubble were transplanted into the field in mid-May, 2014. The experiment design and irrigation regime were the same as that described by Bao et al. (2016). The net photosynthetic rate (Pn) was measured every month after transplantation using an automatic photosynthetic measuring apparatus (GFS-3000; Walz, Effeltrich, Germany). After transplantation for 5 months, the shoot height was measured. Then the shoot was harvested and the shoot DW, the indicators of forage quality, and total P concentration were determined.

Finally, the root sample was collected according to the method reported by Guo et al. (2004) and Xiao et al. (2015) with minor modification. Briefly, soil in an 80 cm × 80 cm quadrate around each single plant was sampled within a range of 60 cm depth using a spade. The thick roots were picked out, and then the soil sample was sieved through a 0.5 mm mesh screen to catch and retain fine roots. After washing with distilled water, the whole root sample was dried in an oven at 80°C for 72 h, then its DW and total P concentration were determined.

### Statistical Analysis

Data were analyzed according to one-way analysis of variance (ANOVA) by SPSS statistical software (Ver. 19.0; SPSS Inc., Chicago, USA) and the significant differences among means were identified by Duncan’s multiple range tests at a significance level of *P* < 0.05. Data were presented as means ± SE (*n* = 9).

## Results

### T_1_ Transgenic Alfalfa Co-overexpressing *ZxNHX* and *ZxVP1-1* Exhibits Enhanced Resistance to Salt and Drought Stress

The T_1_ progeny originated from T_0_ transgenic alfalfa expressing *ZxNHX* and *ZxVP1-1* genes (Bao et al., 2016) were screened for hygromycin resistance (as described in Experimental Procedures). No significant difference of the morphological phenotypes was observed between surviving T_1_ plants and WT. After that, random six surviving T_1_ plants were identified by PCR and RT-PCR methods. The result showed that all of tested plants co-expressed both *ZxNHX* and *ZxVP1-1* genes (data not shown).

**FIGURE 1:**
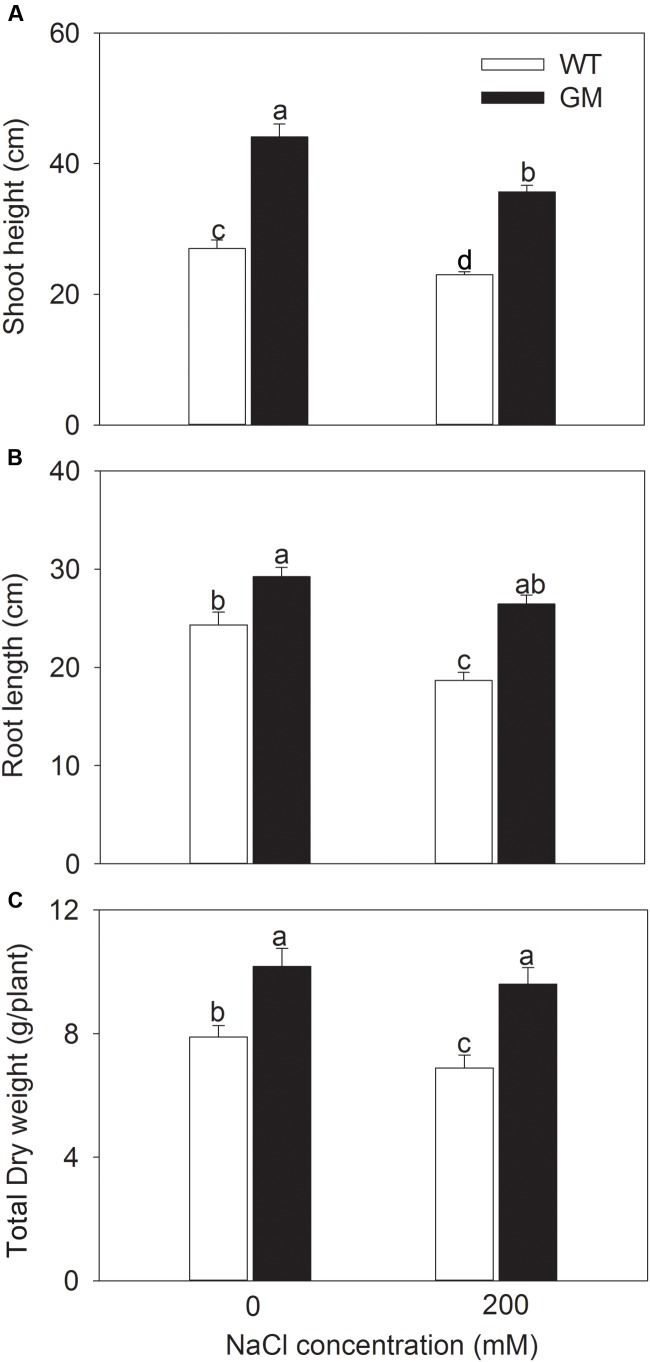
**The growth indicators of wild-type and T_1_ transgenic alfalfa treated with 200 mM NaCl for 20 days.** **(A)** Shoot height; **(B)** root length; **(C)** total DW. Values are the means ± SE (*n* = 9). Different letters after data indicate significant difference (*P* < 0.05) among columns. WT, wild-type plants; GM, T_1_ transgenic alfalfa co-overexpressing *ZxNHX* and *ZxVP1-1*.

**FIGURE 2:**
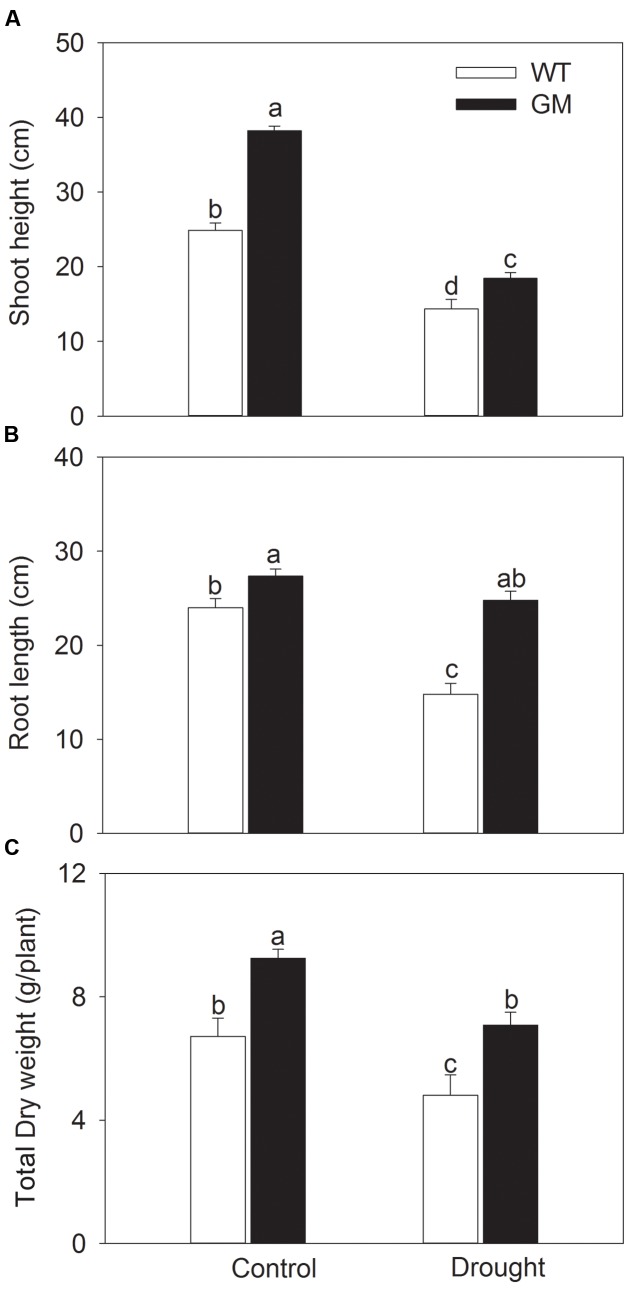
**The growth indicators of wild-type and T_1_ transgenic alfalfa treated with drought (30% of FWC) for 20 days.** **(A)** Shoot height; **(B)** root length; **(C)** total DW. Values are the means ± SE (*n* = 9). Different letters after data indicate significant difference (*P <* 0.05) among columns. WT, wild-type plants; GM, T_1_ transgenic alfalfa co-overexpressing *ZxNHX* and *ZxVP1-1*.

In order to assess the salt and drought tolerance of T_1_ transgenic alfalfa co-overexpressing *ZxNHX* and *ZxVP1-1*, GM and WT plants were cultured under non-stress conditions for 4 weeks, then treated with 200 mM NaCl or drought (30% of FWC) for 20 days. Under normal conditions (no NaCl application), T_1_ transgenic alfalfa exhibited a faster growth than WT; the shoot height, root length, and DW of GM were 63.4, 20.1, and 22.6% higher than for WT plants (**Figure 1**). When treated with 200 mM NaCl, the growth were significantly inhibited in WT while unaffected in GM (except for the shoot height); the shoot height, root length, and DW of GM were significantly higher by 55.1, 40.5, and 39.3%, respectively, than that of WT plants (**Figure 1**). Similarly, the GM showed significantly faster development compared to WT whether treated with drought or not. Under drought stress, the shoot height, root length, and DW of GM were 28.7, 67.7, and 47.5% higher than that of WT plants, respectively (**Figure 2**). Moreover, we also observed that GM accumulated more Na^+^, K^+^, and Ca^2+^ than WT plants under either salinity or drought conditions (**Supplementary Table S1**).

### T_1_ Transgenic Alfalfa Co-overexpressing *ZxNHX* and *ZxVP1-1* Exhibits Improved Adaptation to Phosphate (Pi) Deficiency

**FIGURE 3:**
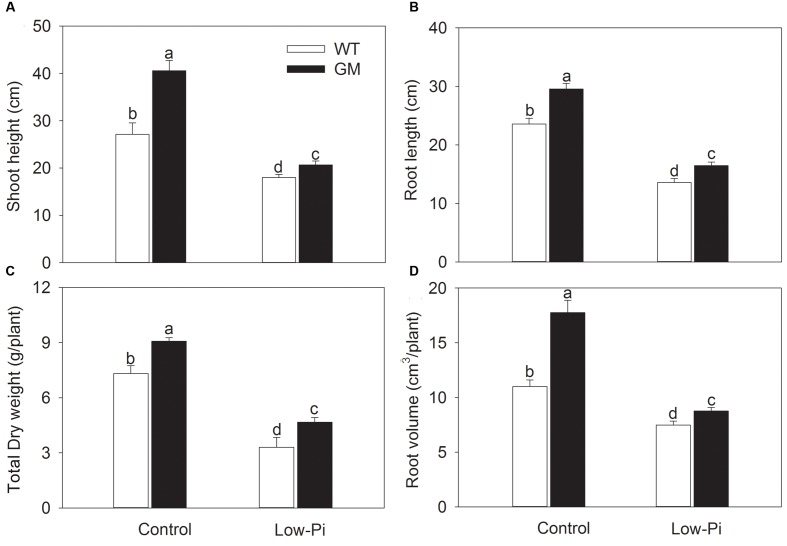
**The growth indicators of wild-type and T_1_ transgenic alfalfa treated with low-Pi (5 μM Pi) for 20 days.** **(A)** Shoot height; **(B)** total dry weight (DW); **(C)** root length; **(D)** root volume. Values are the means ± SE (*n* = 9). Different letters after data indicate significant difference (*P <* 0.05) among columns. WT, wild-type plants; GM, T_1_ transgenic alfalfa co-overexpressing *ZxNHX* and *ZxVP1-1*.

**FIGURE 4:**
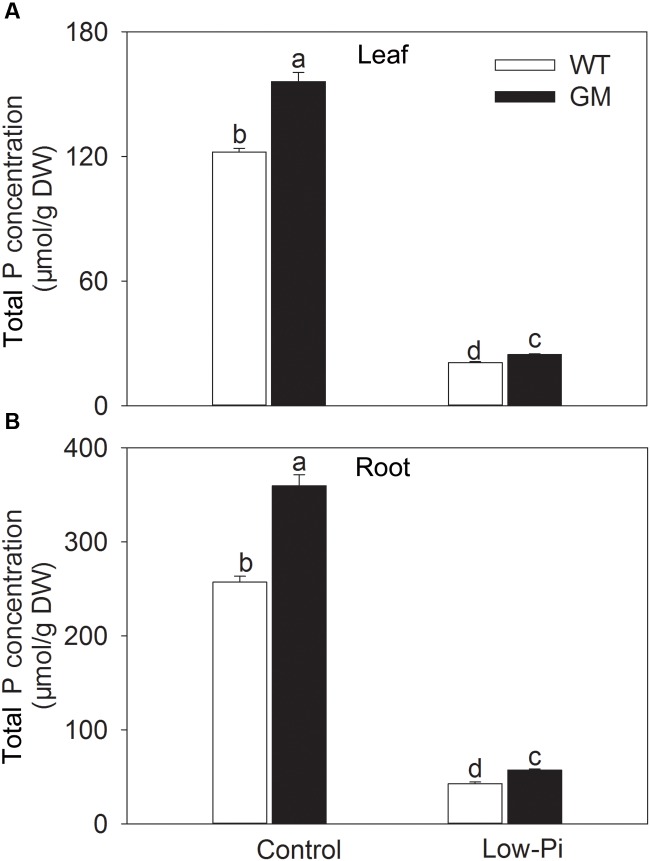
**Total P concentration in leaf **(A)** and root **(B)** wild-type and T_1_ transgenic alfalfa treated with low-Pi (5 μM Pi) for 20 days.** Values are the means ± SE (*n* = 9). Different letters after data indicate significant difference (*P <* 0.05) among columns. WT, wild-type plants; GM, T_1_ transgenic alfalfa co-overexpressing *ZxNHX* and *ZxVP1-1*.

To investigate the growth performance of T_1_ transgenic alfalfa co-overexpressing *ZxNHX* and *ZxVP1-1* in Pi deficiency conditions, four-week-old plants from GM and WT were treated with low-Pi (5 μM) for 20 days. Compared to WT, the GM showed taller and had greater biomass, and especially, developed a more robust root system, whether treated with low-Pi or not. Under low-Pi treatment, the shoot height and DW of GM were 14.6 and 41.2% higher than that of WT plants, respectively (**Figures 3A,B**). Of note, the root length and root volume of GM were significantly higher than that of WT by 25.5 and 61.4% under control, and by 21.3 and 17.1% under low-Pi treatment, respectively (**Figures 3C,D**). The T_1_ transgenic alfalfa accumulated more phosphorus (P) in leaves and roots under normal condition; after being subjected to low-Pi treatment for 20 days, the total P concentrations in leaves and roots of GM were significantly higher by 18.6 and 34.5% than that in WT plants (**Figure 4**).

### T_1_ Transgenic Alfalfa Co-overexpressing *ZxNHX* and *ZxVP1-1* Outperforms WT on Forage Quality in Greenhouse Condition

To assay the forage quality of T_1_ transgenic alfalfa co-overexpressing *ZxNHX* and *ZxVP1-1*, CP, CFI, CF, and CA were determined in shoots of GM and WT plants. As showed in **Table 1**, under control conditions, GM exhibited significantly higher CP and CFI contents than WT by 8.9-20.3% and 18.3-23.7%, respectively. Under 200 mM NaCl, 30% of FWC or 5 μM Pi for 20 days, the CP and CFI contents of GM were 14.6-41.3% and 15.8-20.7% higher than that of WT plants, respectively, though these indicators declined (except for no change of CP in GM under drought) in all plants (**Table 1**). On the other hand, when plants grew under normal conditions, CF and CA showed no difference between GM and WT plants. However, after treated with 200 mM NaCl, 30% of FWC or 5 μM Pi for 20 days, the amounts of CF were reduced by 66.1-73.4% in WT plants, whereas showed no significant change under NaCl treatment or decreased only by 46.2 and 42.9% under drought and low-Pi conditions, respectively; the CA content also decreased less in GM, which was16.3-52.1% higher than for WT plants under various stress conditions (**Table 1**).

**Table 1:** The forage quality indicators of wild-type and T_1_ transgenic alfalfa treated with salt (200 mM NaCl), drought (30% of FWC), and low-Pi (5 μM Pi) for 20 days, respectively.

Experiments	Treatment	Lines	Crude protein (CP) (mg/g DW)	Crude fibre (mg/g DW)	Crude fat (mg/g DW)	Crude ash (mg/g DW)
Salt	Control	WT	166.3 ± 1.8b	209.4 ± 1.7b	23.5 ± 2.8a	97.0 ± 4.3a
		GM	183.0 ± 1.4a	249.3 ± 3.7a	26.3 ± 1.0a	105.7 ± 5.5a
	200 mM NaCl	WT	135.2 ± 1.3d	152.3 ± 1.7d	8.0 ± 0.6b	52.5 ± 1.9c
		GM	154.9 ± 4.2c	176.4 ± 5.1c	25.3 ± 1.0a	79.8 ± 3.8b
Drought	Control	WT	140.8 ± 1.7b	169.9 ± 1.9b	24.8 ± 1.0a	73.5 ± 3.1a
		GM	169.4 ± 4.2a	201.0 ± 2.7a	27.7 ± 1.6a	82.5 ± 5.4a
	30% of FWC	WT	124.4 ± 2.8c	119.5 ± 3.2d	6.6 ± 0.3c	51.9 ± 1.8c
		GM	175.8 ± 4.0a	144.2 ± 3.5c	14.9 ± 1.5b	61.4 ± 1.9b
Low-Pi	Control	WT	161.6 ± 3.1b	198.2 ± 8.1b	25.3 ± 1.0a	109.0 ± 6.5a
		GM	176.0 ± 4.4a	245.2 ± 6.3a	27.2 ± 1.1a	111.6 ± 4.3a
	5 μM Pi	WT	98.4 ± 2.4d	83.0 ± 1.6d	8.2 ± 0.8c	62.5 ± 1.8c
		GM	122.2 ± 9.2c	96.8 ± 4.2c	15.5 ± 1.1b	72.7 ± 1.1b


*Values are the means ± SE (*n* = 9). Different letters after data indicate significant difference (*P <* 0.05) within same column of each individual experiment. WT, wild-type plants; GM, T_1_ transgenic alfalfa co-overexpressing *ZxNHX* and *ZxVP1-1*.*

### T_1_ Transgenic Alfalfa Co-overexpressing *ZxNHX* and *ZxVP1-1* Exhibits Improved Growth and Forage Quality in the Field Conditions

To evaluate the potential productivity of T_1_ transgenic alfalfa co-overexpressing *ZxNHX* and *ZxVP1-1* in semi-arid field conditions, trimmed-plants of GM transgenic line and WT were transplanted into the Yuzhong Experimental Station of Lanzhou University in mid-May, 2014. Compared to WT plants, GM had better growth under field conditions (**Supplementary Figure S1**). After being transplanted for 5 months, the shoot height and shoot DW of GM were 25.1 and 54.1% higher than that of WT, respectively (**Table 2**). Furthermore, GM exhibited significantly higher net Pn than WT plant since the second month of post-transplant. Five months after transplant, Pn of GM was 19.8% higher than that of WT plants (**Supplementary Figure S2**). T_1_ transgenic alfalfa also developed a larger root system. After being transplanted for 5 months, the root DW of GM was 1.8-fold higher than for WT plants, and correspondingly, the root/shoot ratio of GM were significantly higher by 14.1% in comparison with WT plants (**Table 2**). Meanwhile, GM absorbed more Pi with an increased total P concentration in plants (**Table 2**).

**Table 2:** The growth indicators and total P concentration of wild-type and T_1_ transgenic alfalfa after transplanted into field condition for 5 months.

Lines	Shoot height (cm)	Shoot dry weight (DW) (g/plant)	Root DW (g/plant)	Root/shoot ratio	Total P concentration (μmol/g DW)
WT	63.2 ± 1.6b	96.5 ± 3.6b	56.6 ± 2.7b	0.57 ± 0.02b	83.9 ± 1.4b
GM	79.0 ± 1.9a	148.6 ± 7.0a	102.0 ± 3.8a	0.65 ± 0.03a	90.4 ± 1.9a


*Values are the means ± SE (*n* = 9). Different letters after data indicate significant difference (*P* < 0.05) within same column. WT, wild-type plants; GM, T_1_ transgenic alfalfa co-overexpressing *ZxNHX* and *ZxVP1-1*.*

**FIGURE 5:**
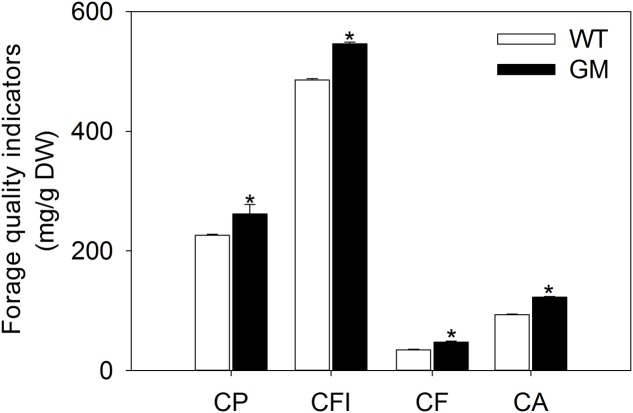
**The forage quality indicators of wild-type and T_1_ transgenic alfalfa after transplanted into field condition for 5 months.** Values are the means ± SE (*n* = 9). Asterisks indicate there is a significant difference (*P <* 0.05) between WT and GM. WT, wild-type plants; GM, T_1_ transgenic alfalfa co-overexpressing *ZxNHX* and *ZxVP1-1*; CP, CP; CFI, crude fiber; CF, crude fat; CA, crude ash.

In order to assess the forage quality of T_1_ transgenic alfalfa co-overexpressing *ZxNHX* and *ZxVP1-1* in the field conditions, the nutrition indicators were measured in shoots. After being transplanted for 5 months, transgenic alfalfa accumulated more CP, CFI, CF, and CA in shoots. The contents of above indicators in GM were significantly higher by 15.9, 12.3, 37.1, and 30.9%, respectively, than in WT plants (**Figure 5**).

## Discussion

### T_1_ Transgenic Alfalfa Inherited Salt and Drought Tolerance from T_0_ Generation

In face of the challenges from salinization and desertification, improving the salt and drought resistance of crops is most efficient and economical way to ensure the food security worldwide (Flowers, 2004; Bartels and Sunkar, 2005; Rozema and Flowers, 2008; Yang et al., 2010). In recent years, the development of molecular biology and transgenic engineering provides tools for creation of new cultivars with enhanced stress tolerance (Hasegawa et al., 2000; Herrera-Estrella, 2000; Yang et al., 2010; Tang et al., 2015; Zhang et al., 2016). Numerous studies have demonstrated that (co-)overexpression tonoplast NHX or/and H^+^-PPase genes is one of the most effective strategies to create transgenic species with enhanced salt and drought tolerance through genetic engineering technology (e.g., Apse et al., 1999; Gaxiola et al., 2001, 2007; Zhao et al., 2006; Liu et al., 2010; Bhaskaran and Savithramma, 2011; Pasapula et al., 2011; Gouiaa et al., 2012; Bao et al., 2014; Bassil and Blumwald, 2014). To develop salt- and drought-resistant forage cultivar, we have co-transferred the tonoplast NHX and H^+^-PPase genes (*ZxNHX* and *ZxVP1-1*, respectively) from a xerophyte *Z. xanthoxylum* into the important legume forage alfalfa and successfully improved the salt and drought tolerance in T_0_ generation transgenic plants (Bao et al., 2016). In the present work, T_1_ generation transgenic alfalfa plants co-overexpressing *ZxNHX* and *ZxVP1-1* also exhibited enhanced salt and drought tolerance. They outperformed WT plants at either plant size or dry matter accumulation under salinity or drought conditions (**Figures 1** and **2**). These results suggest that T_1_ transgenic alfalfa inherited all stress resistant traits from T_0_ generation, and further support that co-overexpression of tonoplast NHX and H^+^-PPase genes from the xerophyte is a feasible way for enhancing salt and drought tolerance of crops.

The better salt and drought tolerance of NHX and H^+^-PPase transgenic plants could be explained as a consequence of increased ion compartmentation into vacuole resulting from increased expression of NHX and H^+^-PPase (Gaxiola et al., 2001, 2007; Leidi et al., 2010; Bao et al., 2014; Yang et al., 2015). Because vacuolar compartmentation of cations (such as, Na^+^ and K^+^) is mediated by NHXs and H^+^-PPase provides the proton motive force for this process as a tonoplast H^+^ pump (Apse et al., 1999; Zhang et al., 2001; Gaxiola et al., 2002; Kronzucker and Britto, 2011; Yamaguchi et al., 2013). This mechanism may contribute to alleviating the toxicity of excessive Na^+^ in the cytosol, maintaining intracellular K^+^/Na^+^ homeostasis, and enhancing vacuolar osmoregulatory capacity (Apse et al., 1999; Blumwald, 2000; Gaxiola et al., 2007; Flowers et al., 2015; Volkov, 2015; Yuan et al., 2015). In our previous study, the co-overexpression of *ZxNHX* and *ZxVP1-1* genes resulted in higher Na^+^, K^+^, and Ca^2+^ accumulation in leaves and roots of T_0_ generation transgenic alfalfa (Bao et al., 2016). This conclusion is further supported by current work, in which T_1_ generation transgenic alfalfa also accumulated more cations under salinity or drought conditions (**Supplementary Table S1**).

### Co-overexpression of *ZxNHX* and *ZxVP1-1* Enhances Phosphorus Nutrition and Productivity of T_1_ Transgenic Alfalfa

Phosphorus (P) is an essential element required for plant growth and development (Ma et al., 2012; Pei et al., 2012; Lv et al., 2015). Phosphate (Pi) is the main form of phosphorus that plant can absorb from soil, and thus is the most limiting factor for plant production in many regions all over the world since the content of available Pi in soil is commonly insufficient (Hinsinger, 2001; Vance et al., 2003; Gaxiola et al., 2011; MacDonald et al., 2011; Bargaz et al., 2016). Therefore, the development of crops with improved phosphorus nutrition will contribute to improvement of crop productivity and reduction of phosphorus fertilizer application (Gaxiola et al., 2011, 2012; Yang et al., 2014). Previous studies showed that *Arabidopsis* tonoplast H^+^-PPase (AVP1) is involved in regulation of Pi uptake of plants. The overexpression of *AVP1* gene in tomato, rice, and *Arabidopsis* have significantly enhanced the growth performance of these species by improving P nutrition in plants (Yang et al., 2007, 2014; Gaxiola et al., 2012). Recent studies demonstrated that up-regulation of the tonoplast H^+^-PPase from other species also increased Pi uptake and thus conferred the low-Pi tolerance in transgenic plants, for example, maize expressing *TsVP1* gene from *Thellungiella halophile* (Pei et al., 2012). Similar results were observed in our study: T_1_ transgenic alfalfa developed larger shoots with higher biomass accumulation under either low-Pi or control conditions (**Figures 3A,B**), which are consistent with increased P accumulation in GM (**Figure 4**); field trial data also showed that T_1_ transgenic alfalfa grew faster with a higher total P concentration than WT after being transplanted into Pi-limiting soil without any P supplement (**Supplementary Figure S1**, **Table 2**). These results suggest that increased P uptake capacity is one of the important contributors for improving productivity of T_1_ transgenic alfalfa, especially under the Pi-limiting conditions. This viewpoint is supported by the data from field trials that both T_0_ (Bao et al., 2016) and T_1_ (**Supplementary Figure S2**) transgenic alfalfa displayed a higher photosynthetic capacity compared to WT, since the P level in plants has a tremendous effect on photosynthetic activity (Jacob and Lawlor, 1991; Dietz and Harris, 1997; Pei et al., 2012), even can improve the salt tolerance of common bean (Bargaz et al., 2016).

Enhancement of Pi uptake in transgenic alfalfa could be explained as a consequence of larger roots, which may result from increased expression of H^+^-PPase in transgenic plants (Gaxiola et al., 2007, 2011, 2012). Li et al. (2005) reported that H^+^-PPase is involved in root development of *Arabidopsis* by regulating the auxin transport and distribution. However, subsequent evidences indicated that AVP1 seems not to be required for auxin transport (Ferjani et al., 2011, 2012; Kriegel et al., 2015). Interestingly, two recent studies revealed a novel function of H^+^-PPase in regulating the long-distance transport of photosynthate from source to sink (particularly, to root) by localizing to the plasma membrane of phloem companion cells (Pizzio et al., 2015; Khadilkar et al., 2016). In the present study, T_1_ transgenic alfalfa developed a larger root system than WT with increased root size and root/shoot ratio under both greenhouse (**Figures 3C,D**) and field conditions (**Table 2**), and thus showed an increased productivity under various conditions (**Figures 1–3**; **Table 2**). These phenotypes are consistent with the observations from other transgenic plants expressing H^+^-PPase (e.g., Park et al., 2005; Yang et al., 2007, 2014; Lv et al., 2008; Bao et al., 2014; Liu et al., 2010). A more robust root system would facilitate the uptake of nutrients and water, which are essential to plant growth in various environments.

### Co-overexpression of *ZxNHX* and *ZxVP1-1* Improves Forage Quality in T_1_ Transgenic Alfalfa

Higher quality is one of most important objectives for forage breeding, since forage quality affects animal’s growth and development, as well as the yield and quality of animal products (Yu et al., 2013; Musco et al., 2016). Therefore, it is necessary to investigate the nutritive value of a potential breeding material before developing it into the new forage cultivar. In this study, the contents of CP and CF, which are important nutritive indicators (Musco et al., 2016), showed higher in T_1_ transgenic alfalfa than in WT under salinity, drought, low-Pi (**Table 1**) and field conditions (**Figure 5**). This may be due to enhanced stress tolerance in transgenic alfalfa, which protects intracellular biochemical synthesis from harsh environments (Zhu, 2001; Flowers et al., 2015). More importantly, transgenic alfalfa accumulated more CP under no-stress conditions (**Table 1**), which could be explained as a consequence of increased nitrogen uptake capacity in transgenic plants by up-expressing H^+^-PPase gene (Paez-Valencia et al., 2013). Our study also showed that transgenic alfalfa deposited more CFI in shoots than WT (**Table 1**, **Figure 5**). This may contribute to the reinforcement of cell wall in transgenic plants, since the CFI is mainly composed of cellulose and hemicellulose, which are two major components of the cell wall (Carpota, 1996; Wolf et al., 2012). The tight cell walls are important for improving the mechanical strength of plants, which allows transgenic alfalfa to grow to greater height and to reduce the loss of cellular water (Darley et al., 2001; Wolf et al., 2012). Moreover, transgenic alfalfa plants also contained more CA than WT plants under various stresses (**Table 1**) and field conditions (**Figure 5**), which may result from the augmented cations (such as, Na^+^, K^+^, and Ca^2+^) accumulation in transgenic alfalfa (Bao et al., 2016). These results indicated that the transgenic alfalfa possesses a higher forage quality.

## Conclusion

The data in this study demonstrate that T_1_ transgenic alfalfa co-overexpressing *ZxNHX* and *ZxVP1-1* genes shows much better tolerance to Pi deficiency, besides the salt and drought. And especially, T_1_ transgenic alfalfa also exhibits improved productivity and higher forage quality. This study laid a solid foundation for developing new alfalfa cultivars with high yield, good quality and wide adaptability on the marginal lands of northern China.

## Author Contributions

PK, A-KB, and S-MW, conceived the study and designed the experiments; PK performed most of the work; TK, Y-QP, ZB, and FW provided the assistance to experiments and data analysis. PK and A-KB wrote the article. S-MW gave valuable suggestions on the article.

## Conflict of Interest Statement

The authors declare that the research was conducted in the absence of any commercial or financial relationships that could be construed as a potential conflict of interest.
