# Supplementary material for: Assessment of Stress Tolerance, Productivity, and Forage Quality in T1 Transgenic Alfalfa Co-overexpressing ZxNHX and ZxVP1-1 from Zygophyllum xanthoxylum
Source: Front Plant Sci. 2016 Oct 27;7:1598. doi: 10.3389/fpls.2016.01598 (PMC5081344; doi:10.3389/fpls.2016.01598)
Supplement: Supplementary file 1 [file Table_1.DOCX]

**Supplementary Table**

**Table S1** Cation concentration in wild-type and T_1_ transgenic alfalfa treated with salt (200 mM NaCl) or drought (30% of FWC) for 20 days, respectively. Values are the means ± SE (*n* = 9). Different letters after data indicate significant difference (*P<*0.05) within same column of each individual experiment. WT, wild-type plants; GM, T_1_ transgenic alfalfa co-expressing *ZxNHX* and *ZxVP1*-*1*.

| Experiments | Treatment | Lines | Na^+^  (mmol/g DW) | | K^+^  (mmol/g DW) | | Ca^2+^  (mmol/g DW) | |
| --- | --- | --- | --- | --- | --- | --- | --- | --- |
|  |  |  | Root | Leaf | Root | Leaf | Root | Leaf |
| Salt | Control | WT | 0.20±0.03c | 0.27±0.03c | 0.34±0.03b | 0.77±0.06b | 0.06±0.01c | 0.32±0.05c |
|  |  | GM | 0.27±0.04c | 0.34±0.02c | 0.50±0.03a | 0.92±0.06a | 0.08±0.01c | 0.49±0.04b |
|  | 200 mM NaCl | WT | 0.89±0.06b | 0.80±0.06b | 0.24±0.03c | 0.62±0.05c | 0.11±0.01b | 0.51±0.04b |
|  |  | GM | 1.21±0.07a | 1.23±0.08a | 0.40±0.02b | 0.83±0.04b | 0.16±0.01a | 0.86±0.06a |
| Drought | Control | WT | 0.17±0.01c | 0.07±0.01c | 0.55±0.04d | 0.63±0.04c | 0.22±0.02b | 0.48±0.06d |
|  |  | GM | 0.19±0.01c | 0.09±0.01c | 1.05±0.08b | 0.90±0.05b | 0.22±0.01b | 0.83±0.12b |
|  | 30% of FWC | WT | 0.27±0.03b | 0.13±0.02b | 0.74±0.05c | 0.71±0.05c | 0.23±0.01b | 0.61±0.03c |
|  |  | GM | 0.35±0.02a | 0.30±0.05a | 1.43±0.09a | 1.29±0.15a | 0.36±0.05a | 1.22±0.14a |
