# Supplementary material for: Assessment of Stress Tolerance, Productivity, and Forage Quality in T1 Transgenic Alfalfa Co-overexpressing ZxNHX and ZxVP1-1 from Zygophyllum xanthoxylum
Source: Front Plant Sci. 2016 Oct 27;7:1598. doi: 10.3389/fpls.2016.01598 (PMC5081344; doi:10.3389/fpls.2016.01598)
Supplement: Supplementary file 2 [file Image_1.PDF]

Supplementary Figures

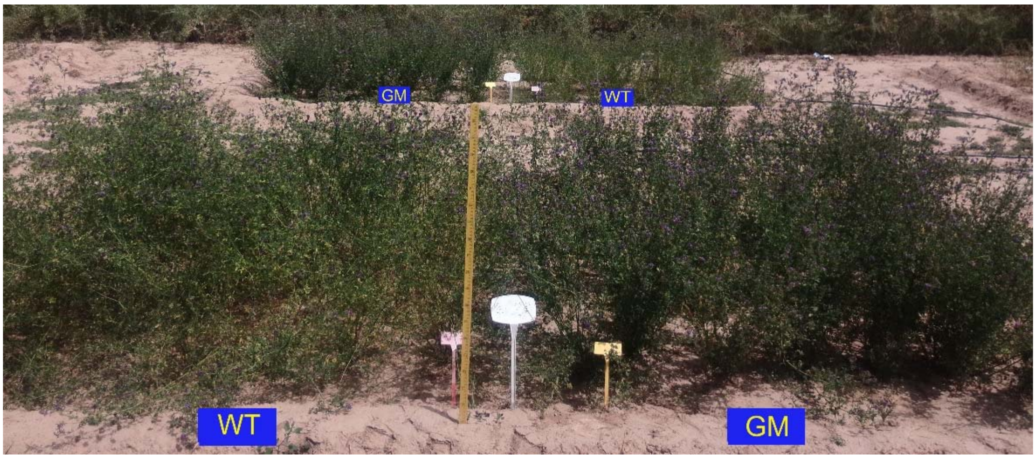

Fig. S1 T_1 transgenic alfalfa under field conditions. T_1 transgenic alfalfa and WT were firstly cultured for 60 days in greenhouse, then the shoots were trimmed away and the remaining plants with 5 cm stubble were transplanted into the field in mid-May, 2014. Representative photograph shows the growth of plants after transplanting for three months under field conditions. WT, wild-type plants; GM, T_1 transgenic alfalfa co-expressing *ZxNHX* and *ZxVP1-1*.

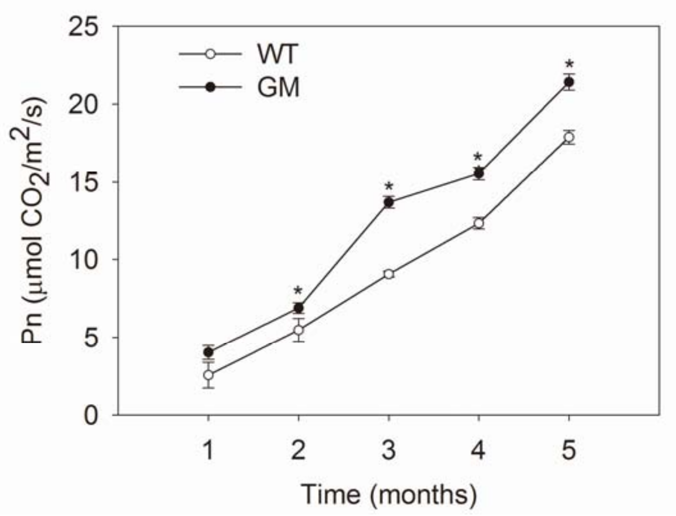

Fig. S2 Net photosynthetic rate (Pn) of wild-type and T1 transgenic alfalfa in the field conditions. Values are the means ± SE (n = 9). Asterisks indicate there is a significant difference (P < 0.05) between WT and GM. WT, wild-type plants; GM, T1 transgenic alfalfa co-expressing *ZxNHX* and *ZxVP1-1*.
